# Shaping Nematic Liquid Crystal Flow States with In-Plane Electric Fields

**DOI:** 10.3390/ma19204355

**Published:** 2026-10-16

**Authors:** Tadej Emeršič, Uroš Tkalec

**Affiliations:** 1 Department of Physics and Materials Science, Faculty of Science, Technology and Medicine, University of Luxembourg, 162a Avenue de la Faiencerie, L-1511 Luxembourg, Luxembourg; 2 Institute of Biophysics, Faculty of Medicine, University of Ljubljana, Vrazov trg 2, 1000 Ljubljana, Slovenia; 3 Department of Condensed Matter Physics, Jožef Stefan Institute, Jamova 39, 1000 Ljubljana, Slovenia; 4 Faculty of Natural Sciences and Mathematics, University of Maribor, Koroška 160, 2000 Maribor, Slovenia

**Keywords:** liquid crystals, microfluidics, flow, electric field, topological defects, dowser texture, interdigitated electrodes

## Abstract

We demonstrate electrical control of flow states in a nematic liquid crystal driven through microfluidic channels. Patterned electrodes generate in-plane fields using a 1 Hz bipolar square-wave drive with peak amplitudes of 3.5 to 9 V. At flow velocities of 50 to 80 $\mu m\;s^{-1}$, these fields reversibly reshape flowing dowser domains, split them into smaller domains, and nucleate new dowser or chiral domains. Field orientation and flow conditions select between domain pairs, individual domains and chiral streams, with nucleation intervals of 0.5 to 2 s under steady flow. Combining the field with pulsating flow produces larger individual domains. These results establish patterned electrodes as localized electrical triggers for controlling nematic flow states, with prospects for integration in optofluidic and lab-on-a-chip devices.

## 1. Introduction

Microfluidics enables precise manipulation of small liquid volumes for analysis, transport, and sorting [1,2]. Its integration with functional materials and diverse actuation mechanisms has produced responsive systems with advanced mechanical, optical, electrical, and biointerfacial properties while providing model environments for confined soft matter far from equilibrium [3,4].

Among the complex fluids explored in microfluidic environments, nematic liquid crystals (LCs) stand out because viscous flow couples with elastic and topological effects [5,6]. Their long-range orientational order competes with hydrodynamic instabilities under confinement and shear, yielding distinct flow-alignment states [7,8,9]. Flowing nematics are governed not only by viscous stresses but also by elastic distortions, surface anchoring, and external fields [10,11]. The director responds through reorientation, backflow, defect formation, and metastable states [12,13,14,15,16]. This interplay makes nematic microfluidics a versatile platform for flow shaping, optofluidics, and studies of topological defects [17,18,19,20].

Recent studies have shown that pressure-driven nematic flow in microchannels can exhibit several distinct flow states and topological structures [5,21,22,23,24], including localized flow-aligned domains bounded by topologically neutral disclination loops [25,26]. The entrapped quasi-planar director field of such domains is closely related to the metastable dowser texture, whose remarkable sensitivity to external perturbations–thickness gradients, flows, and electric fields–has been systematically explored by Pieranski, Godinho, and coworkers [27,28,29]. In our previous work, we demonstrated that these nonequilibrium structures, known as dowser domains, can be stabilized and sculpted by controlling the flow conditions and by applying localized optothermal perturbations [25]. Their size and shape can also be influenced by the channel geometry [26]. Complementary control through patterned surface anchoring has recently been demonstrated in pressure-driven nematic microchannels [30]. These findings raise the important question of whether similarly local, repeatable, and scalable control can also be achieved using electric fields. This possibility is especially appealing because electric fields couple directly to the nematic orientation, are routinely used to create, reconfigure, and steer defect structures and solitonic excitations in nematic layers [31,32,33,34], and can readily be integrated into microfluidic platforms, as demonstrated in electrically programmable nematofluidics, studies of field effects on shear alignment, field-generated nematic microflows, and electrically driven modulation of flow patterns [16,17,35,36]. Related strategies employing surface acoustic waves have recently extended the toolbox of external-field control over channel-confined nematic flows [37].

Here we report on an experimental investigation in which an in-plane electric field is used to manipulate flow-stabilized topologically neutral disclination loops in a homeotropic microfluidic channel. Using patterned interdigitated electrodes, we show that the electric field can reshape, split, reconfigure, and nucleate dowser domains under weak to intermediate flow conditions. These results establish in-plane electrical actuation as a promising route for controlling topological nematic flow states and point toward scalable optofluidic and lab-on-a-chip implementations.

## 2. Materials and Methods

### 2.1. Microfluidic Chip Fabrication and Electric Field Control

**Figure 1:**
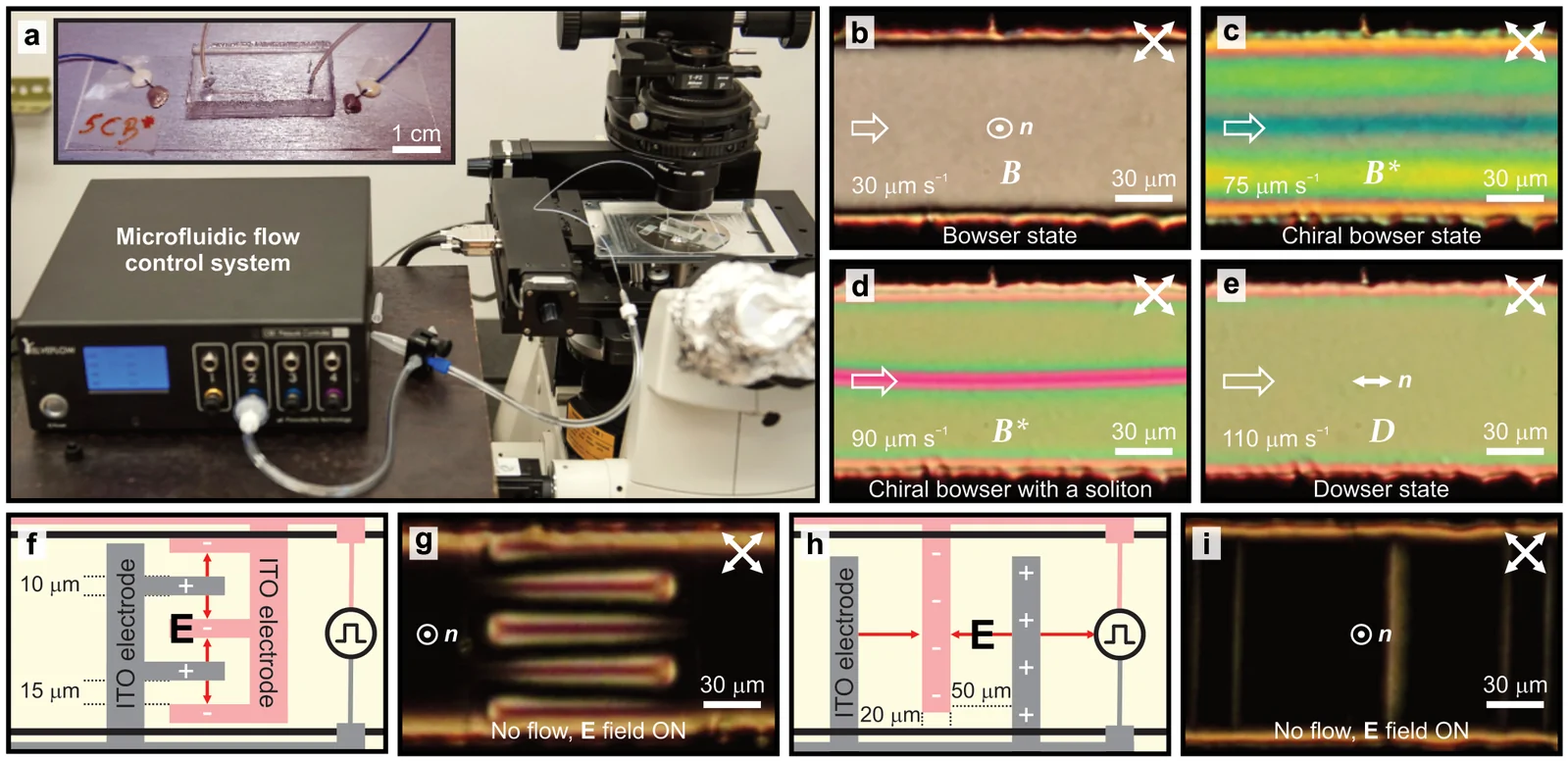
Experimental setup, 5CB flow states, and IDE patterns. (**a**) Pressure controller and PDMS microfluidic chip with ITO electrodes (inset). (**b**–**e**) Bowser *B*, chiral bowser $B^{★}$ without and with a soliton, and dowser *D* states. (**f**,**g**) Transverse-field IDEs with electrode width 10μm and gap 15μm. (**h**,**i**) Longitudinal-field IDEs with width 20μm and gap 50μm. Panels (**g**,**i**) show field-induced birefringence without pressure-driven flow. Open white arrows indicate the flow direction, crossed white arrows mark the polarizer and analyzer axes, and red arrows indicate the electric field axis. The symbols marked n indicate the director orientation. Gray and pink denote the two electrode sets in panels (**f**,**h**). Colors in the micrographs arise from birefringence. The same conventions apply to Figures 2–5.

To fabricate microfluidic channels with indium-tin-oxide (ITO) electrode patterns on the bottom glass substrates, we followed the previously described protocols of standard soft lithography [24,25]. The linear channels have a rectangular cross section with height h≈12μm, width w≈100μm and length L≈20mm (Figure 1a). The channels are assembled from polydimethylsiloxane (PDMS, Sylgard 184, Dow Corning, Midland, MI, USA) reliefs and ITO-patterned glass substrates, which were designed and fabricated by our collaborators (see Acknowledgments). The interdigitated electrodes (IDEs) with high mechanical stability were prepared by a sputtering process on 0.7mm thick glass substrates; the electrode thickness is 300nm. Two IDE geometries were used. For an electric field transverse to the channel, the electrode width and gap were 10 μm and 15 μm, respectively; for an electric field parallel to the channel, they were 20 μm and 50 μm, respectively. The transverse-field array consists of 5 fingers covering a region of approximately 500 μm along the channel, and the longitudinal-field array of 100 fingers covering approximately 8mm; both arrays span the full channel width, and their registration with respect to the channel is shown in Figure 1. A function generator (HMF2525, HAMEG Instruments, Mainhausen, Germany) supplied a continuous symmetric bipolar square-wave voltage U(t) between the electrode sets, and the signal was monitored with a digital storage oscilloscope (TDS1001B, Tektronix, Beaverton, OR, USA). Throughout this article, $\pm U_{0}$ denotes a peak voltage amplitude $U_{0}$, corresponding to a peak-to-peak voltage of $2U_{0}$. The experiments reported here used $U_{0}=3.5$, 5.0, and 9.0V at a frequency of 1Hz. Because the field generated by the IDEs is spatially nonuniform, we report the applied voltage rather than a single electric-field strength. We also note that the two IDE geometries are not matched, so that at a given voltage both the field magnitude and its penetration depth differ between the two configurations: at $U_{0}=3.5\;V$, the nominal in-gap field $E=U_{0}/g$ is $\approx 2.3\times 10^{5}\;Vm^{-1}$ for the transverse-field IDEs and $\approx 0.7\times 10^{5}\;Vm^{-1}$ for the longitudinal-field IDEs, while the field of a periodic array of alternating electrodes decays into the sample over a characteristic length of order Λ/2π set by the period of the potential, $\Lambda =2(w_{e}+g)$, giving ≈8μm (Λ=50μm) and ≈22μm (Λ=140μm), respectively, to be compared with the channel height h≈12μm. A two-dimensional, ion-free electrostatic calculation for the glass/ITO/5CB/PDMS stack (Appendix A) provides a reference for the spatial field distribution. The high-permittivity nematic layer channels the field: at the experimental voltages, the computed transverse-field gap-center magnitude exceeds the uniform-layer reorientation field scale throughout the channel height, whereas the longitudinal-field gap-center magnitude varies only weakly with height. This calculation does not determine the time-dependent field after ionic screening. Quantitative comparisons are therefore made only within a given electrode geometry, and comparisons between the two configurations remain qualitative. In addition to the electric field application, the ITO layer efficiently absorbs infrared (IR) laser light and thus provides precise control over the local heating of the flowing nematic. Before starting the flow experiments, the channel walls were chemically functionalized with a 0.2wt% aqueous solution of dimethyloctadecyl[3-(trimethoxysilyl)propyl]ammonium chloride (DMOAP, ABCR, Karlsruhe, Germany), and finally dried in a stream of hot air to render the PDMS surface and glass substrate hydrophobic. This surface coating is known to induce strong homeotropic alignment of the single-component NLC 4-cyano-4’-pentyl-1,1’-biphenyl (5CB, Synthon Chemicals, Bitterfeld-Wolfen, Germany) at the interfaces [5]. The functionalized channels were filled with 5CB in the isotropic phase (above 35 °C) and then gradually cooled down to the nematic phase at room temperature within a couple of minutes.

### 2.2. Flow Control and Optical Characterization

The channel-confined nematic flow is driven by a piezoelectric pressure control system (OB1 MK3, Elveflow, Paris, France), with driving pressure differences in the range of 80 to 215 mbar in the reported experiments. The volumetric flow rate, estimated from the controller calibration, was varied between 0.1 and $0.6\;\mu L\;h^{-1}$, corresponding to mean flow velocities of approximately 20–$140\;\mu m\;s^{-1}$ in the channel cross section. Because the effective viscosity of the nematic depends on the director configuration, we use the tracked translational velocity of the domains and defect structures as the operational measure of the flow velocity throughout this work. The translational velocities of the observed dowser domains and defect structures, determined by tracking them in the recorded movies, ranged from 10 to $100\;\mu m\;s^{-1}$; the difference with respect to the mean flow velocity reflects the drag of the disclination loop and the position of the domain within the Poiseuille profile [24,25]. We used the built-in flow profile routines of the controller, which precisely maintained the applied pressure. Poiseuille flow of 5CB in rectangular microchannels is strictly laminar, with an estimated Reynolds number Re=ρvh/η of approximately $2\times 10^{-6}$ to $2\times 10^{-5}$ (taking the density $\rho \approx 1.02\times 10^{3}\mathrm{kg}m^{-3}$ and an effective dynamic viscosity η≈50mPa·s). The Ericksen number Er=γvh/K, defined with the full channel height *h* as the length scale, varies roughly between 2 and 20 (taking K≈5.5pN in the single elastic constant approximation, the average value used for consistency with our previous studies [24,25], and the rotational viscosity γ≈81mPa·s) [8]. Using the larger room-temperature splay or bend elastic constants would give smaller values of Er; the quoted range should therefore be read as an approximate estimate based on the stated effective value of *K*. 5CB has positive dielectric anisotropy, Δε≈11.5 at room temperature and low frequency, so the long axes of the molecules align along the direction of the applied electric field.

The flow was observed in transmission between crossed polarizers using an inverted microscope (Eclipse Ti-U, Nikon, Tokyo, Japan) with CFI Plan 10× and 20× objectives. Channels were oriented at $45^{{}^{\circ}}$ to the polarizers to enhance birefringence contrast. A laser-tweezers setup built around the microscope used a linearly polarized IR fiber laser at 1064nm. Computer-controlled acousto-optic deflectors (Tweez200si, Aresis, Ljubljana, Slovenia) positioned the diffraction-limited beam, whose power at the sample was varied from 20 to 200mW. IR laser pulses were used to prepare dowser domains, and their subsequent electrical manipulation was observed with the laser switched off. Movie S1 includes preparation pulses at about 3 and 9 s, and Movie S3 at the beginning and at about 8 s. The associated transient isotropic regions are distinct from the electric-field-induced response. Full-HD color videos were recorded at 25 frames per second using a CMOS camera (EOS 750D, Canon, Tokyo, Japan) and analyzed with VideoMach. All measurements were performed at $T={\left(23\pm 2\right)}^{{}^{\circ}}C$.

## 3. Experimental Results and Discussion

In this article, we present and study the dynamics of flowing dowser domains inside a PDMS microfluidic chip using a combination of flow and electric field manipulation. A thin film of interdigitated ITO electrode (IDE) patterns on the bottom glass substrate was used for generating an in-plane electric field. The flow of the LC inside the homeotropic PDMS microfluidic channel was precisely controlled by the piezoelectric pressure controller, as shown in Figure 1a. The various flow regimes of 5CB inside a homeotropic microchannel were already identified and described in our previous study [24]. First, in the weak flow regime, the uniform homeotropic director field becomes slightly bowed towards the direction of flow, which causes the appearance of birefringent colors under crossed polarizers (Figure 1b); this state is therefore named the “bowser state” (*B*). The weak flow regime is followed by the medium flow regime, where the *B* state gradually becomes unstable and breaks the symmetry via a second-order transition into the chiral bowser state ($B^{★}$) of left- and right-handed regions, with a flexible nonsingular soliton forming wherever oppositely handed regions meet, typically in the middle of the channel (Figure 1c,d). With increasing flow velocity, the director field undergoes a discontinuous first-order transition into a flow-aligned state known as the dowser (*D*) state. The director field of this flow-oriented phase is completely aligned horizontally in the midplane (Figure 1e). Figure 1f,h shows schematics of the IDEs on the bottom glass substrate of the microfluidic channel for generating an AC in-plane electric field perpendicular to and along the channel, respectively. In the absence of pressure-driven flow, the electric field induces distortions of the director field around the electrodes, which are visible as birefringent colors under crossed polarizers in the transverse-field (Figure 1g) and longitudinal-field (Figure 1i) configurations. The first visible optical modulation at the electrode edges appears at approximately ±1.5V.

In our previous study, we have shown the existence of a critical velocity below which all dowser domains are unstable and gradually shrink over time until they disappear [25]. When the critical velocity is reached, only the domains larger than the critical size become stable; otherwise, they shrink and annihilate. Here, we investigate the impact of in-plane electric field modulation on the nematic flow and on flowing dowser domains at velocities slightly greater than critical.

### 3.1. In-Plane Electric Field Perpendicular to the Flow

**Figure 2:**
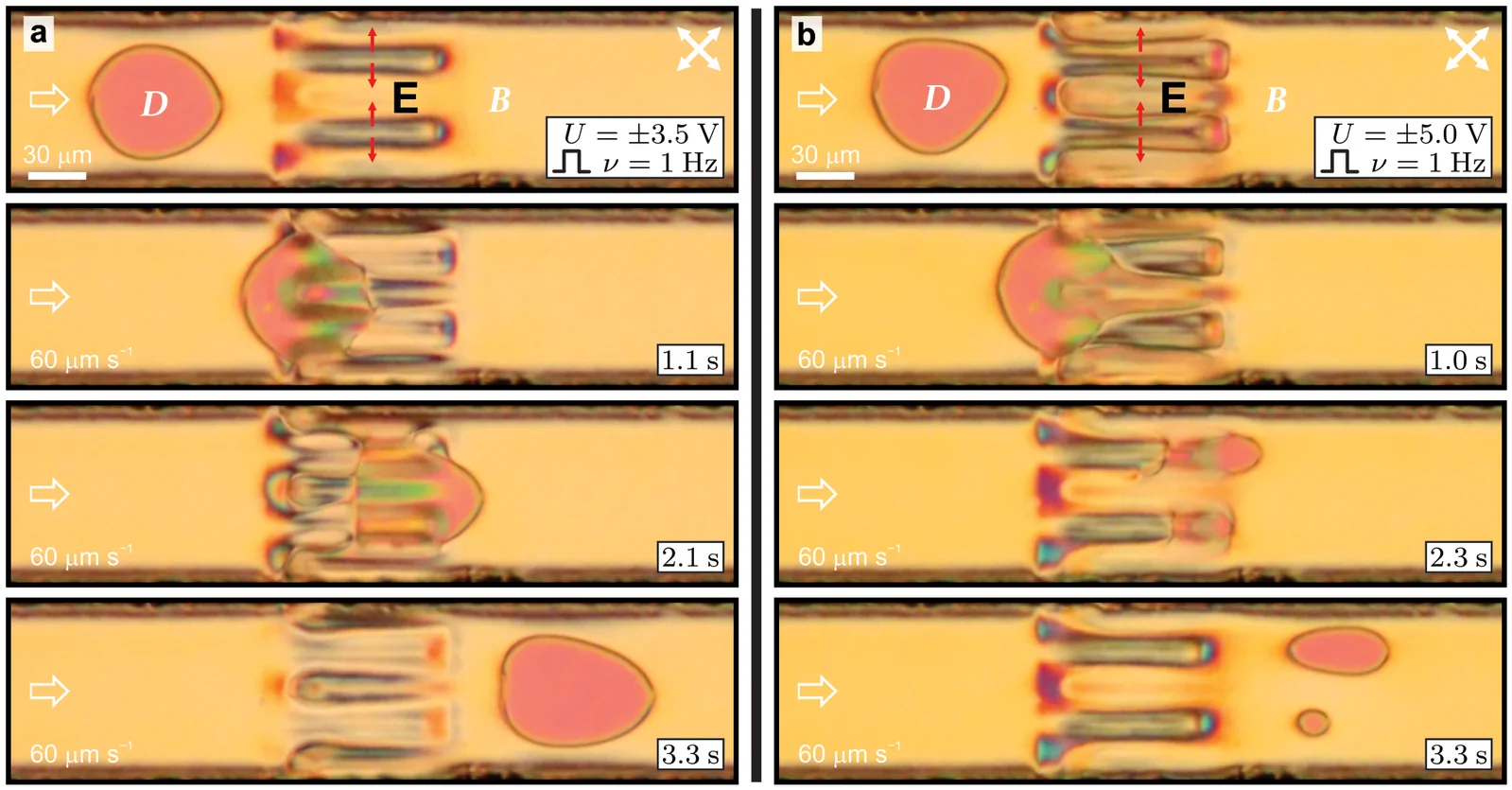
Transverse-field manipulation of a dowser domain at $v=60\mu ms^{-1}$ and 1Hz. (**a**) At ±3.5V, the domain is slightly reshaped and changes birefringence. (**b**) At ±5.0V, it splits into two smaller domains. The passage times through the IDEs are comparable at the two amplitudes.

First, we investigated an in-plane field perpendicular to the channel and flow. Figure 2a shows a dowser domain in the weak-flow regime, moving at $60\;\mu m\;s^{-1}$ through a square-wave field U(t)=±3.5V at 1Hz. The spatially nonuniform electrode field perturbs the director, and elastic coupling transmits the resulting distortion through the channel, changing the domain shape and optical appearance. As a control, passages over the same IDEs at the same velocities with the field off, or at low voltages $U_{0}\leq 2$ V, produced no detectable reshaping of the passing dowser domains. The field-off passages constrain the influence of the 300-nm electrode steps and possible ITO/glass anchoring differences. The absence of a detectable domain response at low voltage does not imply the absence of the localized director distortions visible near electrode edges at approximately ±1.5 V. After leaving the field region, the domain recovers its unperturbed shape; traveling above the critical velocity, it then continues to grow until it reaches the channel walls, after which it expands along the channel, following the field-free supercritical dynamics described in Ref. [25]. The domain recovers its characteristic shape on the scale of one drive period after leaving the electrode region while continuing to grow. Figure 2b shows a domain at the same velocity under ±5.0V at 1Hz. The higher voltage completely reshapes the domain and splits it into two unequal daughter domains. Each is typically below the critical size, and, therefore shrinks and annihilates. The size difference could arise from asymmetry in the flow profile, a slightly biased flow velocity, or IDE position. Across our archive of 8 recordings, splitting at ±5.0 V and $60\;\mu m\;s^{-1}$ was observed in approximately one third of the analyzed passes, whereas at ±3.5 V reshaping without splitting was observed in every analyzed pass. These fractions describe the recorded sample and are not estimates of device-independent success probabilities. The loops passed through the IDEs in approximately 3s at both amplitudes. For the representative passage in Movie S1, centroid tracking in the unperturbed intervals 4.0–5.0 s before and 8.0–8.8 s after the electrodes gives a post/pre velocity ratio of approximately 1.00, remaining within 2% of unity across the tested segmentation thresholds. This relative comparison is independent of the spatial calibration and excludes the later laser-preparation pulse at about 9 s. It supports no resolvable velocity change during this passage, without establishing a general absence of field-induced flow.

**Figure 3:**
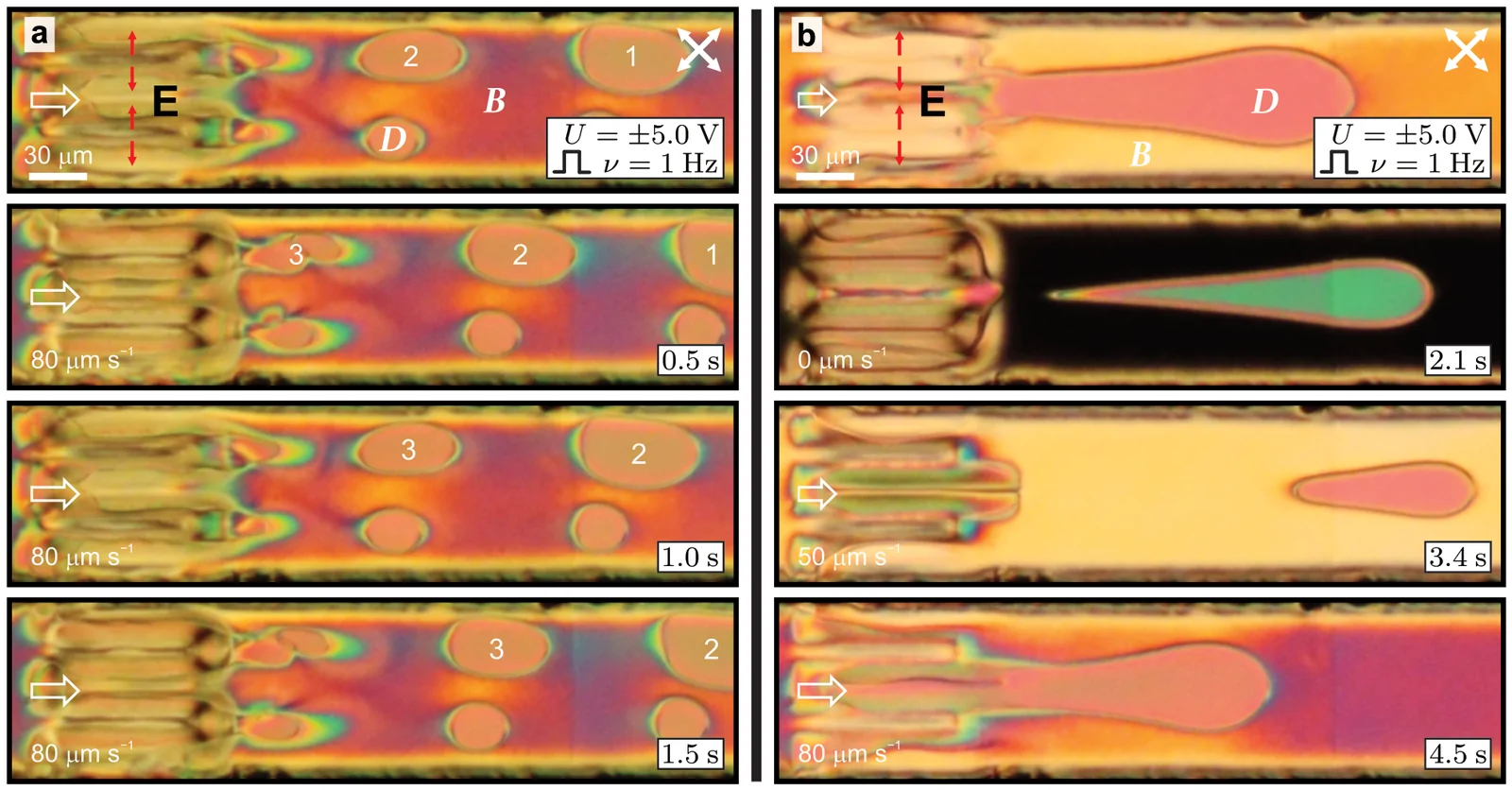
Transverse-field nucleation of dowser domains at ±5.0V and 1Hz. (**a**) At $v=80\;\mu m\;s^{-1}$, two domains nucleate every 0.5s. (**b**) With flow modulated between 0 and $80\;\mu m\;s^{-1}$ over 5s, a single domain nucleates. Numbers 1–3 label successive domain-pair nucleation events.

We investigated the impact of the same square waveform modulation of the in-plane electric field, with an amplitude of ±5V and a frequency of 1Hz, also at higher flow velocities, with the flow either steady at $v\approx 80\;\mu m\;s^{-1}$ or modulated between 0 and $80\;\mu m\;s^{-1}$ (pulsating-flow protocol). We found that a velocity around $80\;\mu m\;s^{-1}$, which in our channels lies close to, but still below, the threshold of the $B\to B^{★}$ transition, leads to domain creation, as shown in Figure 3a. A sufficiently strong medium flow of the LC in a homeotropic microfluidic channel tends to orient the LC molecules into the intermediate chiral state $B^{★}$, which typically arises before the *B*-to-*D* transition. In this near-threshold flow, where the channel in the absence of the field remains in the *B* state, the AC in-plane electric field nucleates flow-aligned states bounded by disclination loops–dowser domains. Production occurs from a distorted area of the director field at the end of the IDE pattern. After creation, the domain loops evolve in the flow as expected and as already reported in our previous study [25]. Under this configuration of the IDEs, with the AC in-plane electric field perpendicular to the flow, two small domains are produced simultaneously every 0.5s. The nucleated domains are of different sizes, which could have the same explanation as in the previous example of the domain splitting mechanism. With this transverse-field geometry and the same electric parameters, the production of a single dowser domain was achieved using pulsating flow, as shown in Figure 3b. Here, increasing the flow velocity aligns the molecules along the flow direction into the *D* state, starting at the end of the IDE pattern. The subsequent decrease of the velocity tends to align the molecules back into the initial homeotropic state, which is unfavorable for dowser domains. Therefore, the large domain shrinks into a smaller elongated shape with the longer axis along the channel. Figure 3b shows the production of such a flowing dowser state, which typically occurs for a sinusoidal modulation of the flow velocity between 0 and $80\;\mu m\;s^{-1}$ with a period of 5s. One can observe that with the pulsating flow, the production of domains is slowed down and reduced to a single domain, which is, however, significantly larger and elongated along the channel in the direction of the nematic flow.

### 3.2. In-Plane Electric Field Along the Channel

**Figure 4:**
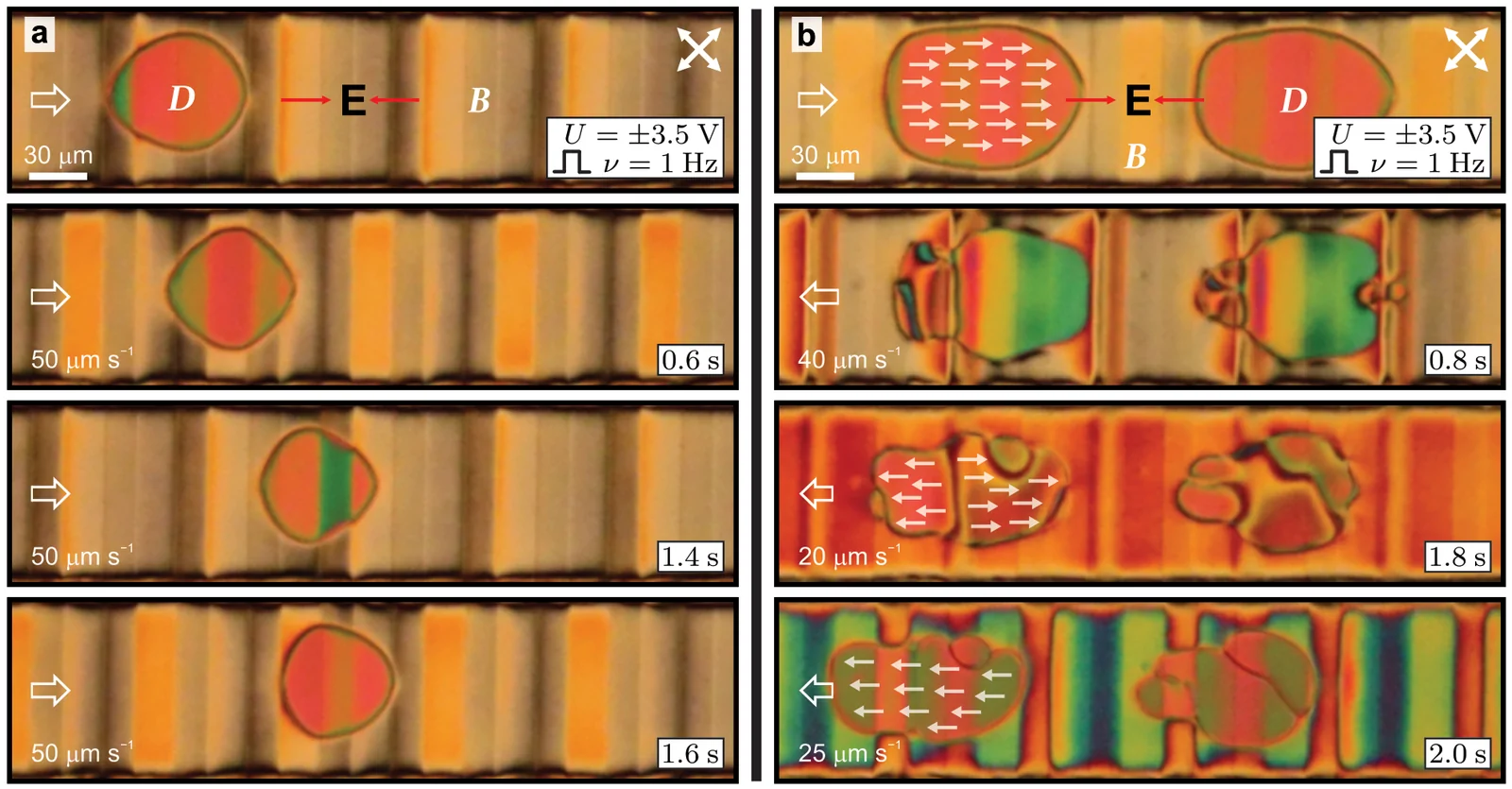
Longitudinal-field reshaping at ±3.5V and 1Hz. (**a**) At $v=50\;\mu m\;s^{-1}$, the domain alternately elongates and contracts. (**b**) Under alternating flow, reversal reorients the domain and creates surface defects and realignment fronts, while the field reshapes the transient director configuration. Small white arrows within the domains in panel (**b**) schematically indicate the local in-plane director orientation.

We also investigated an in-plane field along the homeotropic channel. At ±3.5V and 1Hz, switching the field parallel and antiparallel to the flow reorients the LC around the IDEs, seen as changing birefringent colors (Figure 4a). The resulting distortion modifies the shape and birefringent colors of a dowser domain in the channel midplane. The image sequence is suggestive of a polarity-correlated response: at $50\;\mu m\;s^{-1}$, the domain alternately elongates and contracts during the voltage cycle. We note, however, that at this velocity, the domain crosses one 70μm finger period of the electrode array in about 1.4 s, which is comparable to the 1 s drive period, so the temporal (drive) and spatial (electrode-passing) modulations cannot be fully separated in this sequence. The polarity-correlated interpretation should therefore be regarded as tentative, pending experiments at different drive frequencies. Figure 4b shows two domains in an alternating flow, $-60\leq v\leq 60\;\mu m\;s^{-1}$, with a period of 4.0s. Each flow reversal reorients the domains and creates surface point defects and realignment fronts [25]. The field further reshapes the entrapped director into transient subdomains with different orientations, which realign with the flow as the velocity increases.

**Figure 5:**
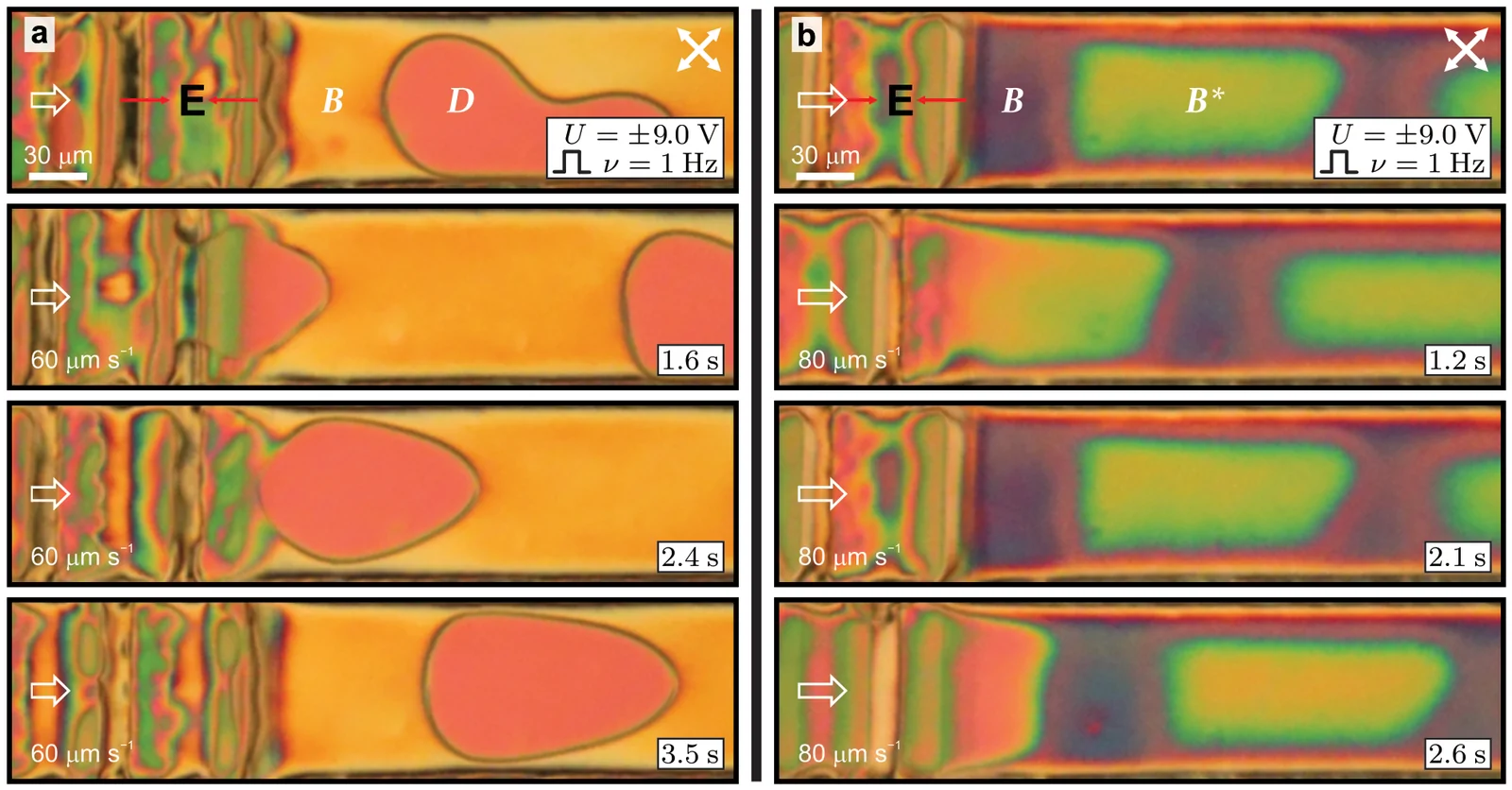
Longitudinal-field nucleation at ±9.0V and 1Hz. (**a**) At $v=60\mu m\;s^{-1}$, dowser domains nucleate every 0.8s. (**b**) At $v=80\mu m\;s^{-1}$, chiral domains are emitted approximately every 2 s. The $B^{★}$ packets are bounded by flexible nonsingular soliton walls separating them from the achiral *B* background.

In Figure 5a, we show that with an AC in-plane electric field parallel and antiparallel to the flow, it is also possible to achieve the production of single dowser domains within a constant pressure-driven nematic flow. For this purpose, a higher driving voltage (±9V) of square waveform needs to be applied at a nematic flow velocity around $60\;\mu m\;s^{-1}$. The domains nucleated in the longitudinal-field configuration (Figure 5a) are considerably larger than those nucleated in the transverse-field configuration (Figure 3a): measured ≈200 μm downstream of the electrode region, their areas are $A\approx 8.3\times 10^{3}\;\mu m^{2}$ and $A\approx 1.5\times 10^{3}\;\mu m^{2}$, respectively, as measured from the recorded sequences. These values characterize representative events rather than statistically established size distributions. Here, the creation of a domain typically takes less than a second. As seen in the images, the higher applied voltage also creates a more visible distortion around the IDE patterns. At a higher flow velocity of $80\;\mu m\;s^{-1}$–again close to, but still below, the $B\to B^{★}$ threshold, so that the channel in the absence of the field remains in the *B* state–and the same peak voltage amplitude of ±9V, one can produce chiral $B^{★}$ domains and form their steady stream inside the *B* state, as shown in Figure 5b. Successive packets are emitted at intervals of approximately 2 s. Tracking seven consecutive packets in Movie S4 gives six intervals of 1.96–2.04 s, with a mean of 2.00 s at the 0.04 s frame spacing. The nucleated $B^{★}$ packets are regions of flow-induced chiral symmetry breaking, in which the director escapes out of the flow plane with a well-defined handedness; they are bounded by flexible nonsingular soliton walls that separate them from the surrounding achiral *B* state [24]. In exploratory frequency scans, successive packets could appear with alternating handedness, suggesting a correlation with the drive, but a systematic correlation between packet handedness and field polarity cannot be established from the present recordings and remains an open question.

The observed phenomenology can be rationalized by the interplay of dielectric, elastic, and hydrodynamic torques. The quadratic dielectric torque is a plausible primary trigger. The nominal estimates $U_{0}/g$ span approximately 0.7–$3.3\times 10^{5}\;V\;m^{-1}$ over the reported geometries and voltages, comparable to or above the uniform, strongly anchored homeotropic-layer field scale $E_{\mathrm{th}}=\left(\pi /h\right)\sqrt{K/(\varepsilon_{0}\Delta \varepsilon )}\approx 6\times 10^{4}\;Vm^{-1}$. Appendix A gives the more appropriate spatially nonuniform, unscreened reference fields: the transverse-field gap-center magnitude exceeds this scale throughout the channel height, whereas the longitudinal-field value is below it at ±3.5 V and above it at ±9 V. This comparison is consistent with the different responses but is not a calculation of the nucleation threshold in a flowing, metastable texture. The recorded electrode-region intensity has a pronounced component near twice the drive frequency. This is consistent with an even-in-field or reversal-associated response but does not uniquely identify dielectric actuation: for an ideal symmetric square wave, $U{\left(t\right)}^{2}$ is constant between reversals, and optical transmission is itself a nonlinear function of director orientation. Because the dielectric torque is quadratic in E, it is invariant under field reversal and cannot by itself explain a polarity-dependent response. Such a response requires an additional contribution, for example, linear flexoelectric coupling [38] and/or an electrokinetic effect. Since 5CB has a positive dielectric anisotropy, the dielectric contribution tends to align the director near the bottom substrate with the local in-plane field axis. The resulting distortion is transmitted through the 12μm thick channel by elasticity, locally frustrating the homeotropic (bowser) background. When the imposed distortion exceeds the barrier separating the metastable states of the flow diagram, the field acts as a repeatable, lithographically defined trigger for the first-order transition into the flow-aligned state, in close analogy to the localized optothermal quench used in our earlier work [25]. We further note that the quasi-planar director field entrapped within a dowser domain carries a flexoelectric polarization collinear with the dowser field, so that in-plane electric fields couple to it already in first order [28,39,40]. Two distinct electrokinetic effects could additionally contribute: electro-osmosis linear in E, as discussed for the dowser texture by Pieranski and Godinho [39], and LC-enabled electro-osmosis, which is quadratic in E and arises from space-charge separation in distorted director regions [41]; our present data cannot discriminate between them, and since our channels intentionally contain single-component 5CB without tracer particles, a direct velocimetry of field-induced flows is left for dedicated tracer-seeded cells. Such linear coupling, together with the quadratic dielectric torque and possible electro-osmotic contributions [39], provides several channels through which low-frequency in-plane fields can reshape and reorient the entrapped director field, consistent with the pronounced sensitivity of the domains observed in Figure 2 and Figure 4.

The timing of the nucleation events suggests a role for field reversals and advection. The 0.5 s interval between domain-pair events in Figure 3a matches the half-period of the drive, but matching intervals alone do not establish absolute phase locking. The approximately 2 s packet-emission interval in Figure 5b spans two drive periods. The 0.8 s interval in Figure 5a is not an integer multiple of the half-period. A possible additional time scale is the clearance of the nucleation region by advection: taking a characteristic length of 50μm and a velocity of $60\;\mu m\;s^{-1}$ gives about 0.8 s. This order-of-magnitude agreement is a plausible interpretation rather than proof of a rate-limiting mechanism; it also distinguishes the formation interval from the size of a domain measured farther downstream.

At the low drive frequency used here, ionic charge redistribution can modify the electric field. For typical conductivities $\sigma \approx 10^{-8}$–$10^{-7}\;S\;m^{-1}$ and $\bar{\varepsilon }\approx 11$, the bulk charge-relaxation time $\tau_{q}=\varepsilon_{0}\bar{\varepsilon }/\sigma$ is approximately 1–10 ms. This time is distinct from electrode double-layer charging and, by itself, does not give the fraction of a half-cycle for which the bulk field remains unscreened [42]. Estimates of double-layer charging on the order of 0.2–0.8 s depend on ionic transport and interfacial properties that were not measured here; they suggest a possible competition with the 0.5 s half-period and with the director-relaxation time $\tau_{d}=\gamma h^{2}/\left(K\pi^{2}\right)\approx 0.2$ s. Reversal-associated transients are therefore a plausible contribution, analogous to transient ion-mediated switching in bistable boundary-layer displays [43], but the instantaneous screened field is not determined by the present data. In exploratory scans at $U_{0}\approx 5\;V$, splitting and nucleation were observed within a low-frequency window of approximately 0.3–10 Hz; at 50–500 Hz no effect on the defect loops was detectable, while below approximately 0.3 Hz domain production ceased. A systematic sweep at fixed RMS amplitude, with synchronized current and optical measurements and small DC offsets, would help distinguish transient, linear, and quadratic contributions.

The conductivity of the 5CB batch and the cell current were not measured. The DMOAP alignment layer was not characterized as an electrically blocking layer, so electrochemical contributions at ±9 V and 1 Hz cannot be excluded. No bubble formation, electrode darkening, or noticeable drift of the response thresholds was observed during repeated sessions, but these visual observations do not establish long-term electrochemical stability.

A quantitative separation of these contributions would require numerical modeling of the coupled director and velocity fields in the actual electrode geometry, for which established nematodynamic approaches are available [35,36,44,45,46,47,48,49,50], and it remains an interesting task for future work.

Table 1 summarizes all field-induced responses observed in this work, organized by electrode orientation, drive amplitude, and flow protocol.

## 4. Conclusions

In summary, we have demonstrated experimentally that low-frequency AC in-plane electric fields, generated by interdigitated ITO electrodes patterned on the bottom substrate of a homeotropic microfluidic channel, provide a versatile and repeatable tool for manipulating topologically distinct flow states in a pressure-driven nematic LC. Depending on the electrode orientation, field amplitude, and flow conditions, the electric field can (i) reversibly reshape flowing dowser domains and modulate their birefringence at ±3.5V, (ii) split individual domains at ±5V, (iii) periodically elongate and shrink domains when applied along the channel, and (iv) nucleate dowser or chiral $B^{★}$ domains, using $U_{0}=5.0\;V$ for the transverse IDEs and $U_{0}=9.0\;V$ for the longitudinal IDEs. Nucleation proceeds from the field-distorted end of the electrode pattern and can be tuned from periodic domain pairs to single, larger domains by combining field modulation with pulsating flow. The electrodes play a role analogous to the focused laser pulses of our previous experiments [25], with the qualification that fixed electrodes cannot be repositioned: the analogy is limited to the localized and repeatable triggering of director distortions without a focused optothermal preparation pulse, while the electrical counterpart of laser-beam steering would be an individually addressable multi-electrode array, a natural next step. Compared with the static, geometry-induced reshaping and splitting of domains imprinted permanently in the channel design [26], electrical actuation performs similar operations dynamically and reversibly in a channel of fixed geometry, with the type of operation selected by the voltage amplitude, frequency, and timing of the drive. The demonstrated operation is repeatable under the reported conditions; extensions toward large-area, multiplexed, and sensing applications in optofluidic modulators, microcargo transport and lab-on-a-chip devices [18,19,20] remain prospects that will require dedicated addressable electrode arrays and systematic electrical characterization. A complementary direction is to combine electrode addressing with structured (patterned) surface alignment: an anchoring pattern could imprint a static director template defining preferential nucleation, pinning, and routing sites for dowser domains [30], while the electrodes would supply the temporal, on-demand control demonstrated here. The potential of pre-programmed alignment in responsive LC media is well illustrated by the programmable, flow-free transport of microcargo achieved in photoresponsive cholesteric films [51]. Quantitative modeling and optimized electrode and material designs are natural next steps.

## Figures and Tables

**Table 1:** Summary of the observed field-induced responses of the nematic flow states. $U_{0}$ denotes the peak amplitude of the 1 Hz bipolar square-wave drive, unless stated otherwise.

Configuration (Width/Gap)	$U_{0}$ (V)	Flow (μm $s^{-1}$)	Observed Response	Figure
Transverse field (10/15 μm)	3.5	60, steady	Reversible reshaping of dowser domain; birefringence modulation	Figure 2a
Transverse field	5.0	60, steady	Splitting into two subcritical daughter domains, which shrink and annihilate	Figure 2b
Transverse field	5.0	≈80, steady (near $B\to B^{★}$ threshold)	Nucleation of dowser-domain pairs, one pair per 0.5 s	Figure 3a
Transverse field	5.0	0–80, pulsating (5 s period)	Nucleation of a single, larger elongated dowser domain per flow cycle	Figure 3b
Longitudinal field (20/50 μm)	3.5	50, steady	Periodic elongation and contraction of passing domain	Figure 4a
Longitudinal field	3.5	−60 to +60, alternating (4 s period)	Domain reorientation at each flow reversal; transient subdomains; surface point defects and realignment fronts	Figure 4b
Longitudinal field	9.0	60, steady	Nucleation of single dowser domains every 0.8 s	Figure 5a
Longitudinal field	9.0	80, steady	Steady stream of chiral $B^{★}$ packets, one every 2 s	Figure 5b
Either	≤2.0	50–80	No detectable domain reshaping (low-voltage control)	—

Exploratory scans (not shown): splitting was first observed at ±4.0V; no detectable domain reshaping occurred for $U_{0}\leq 2\;V$ in the tested flow range, and no effect on the defect loops was detected at 50–500 Hz and ±5.0V. Domain production ceased below approximately 0.3 Hz.

**Figure A1:**
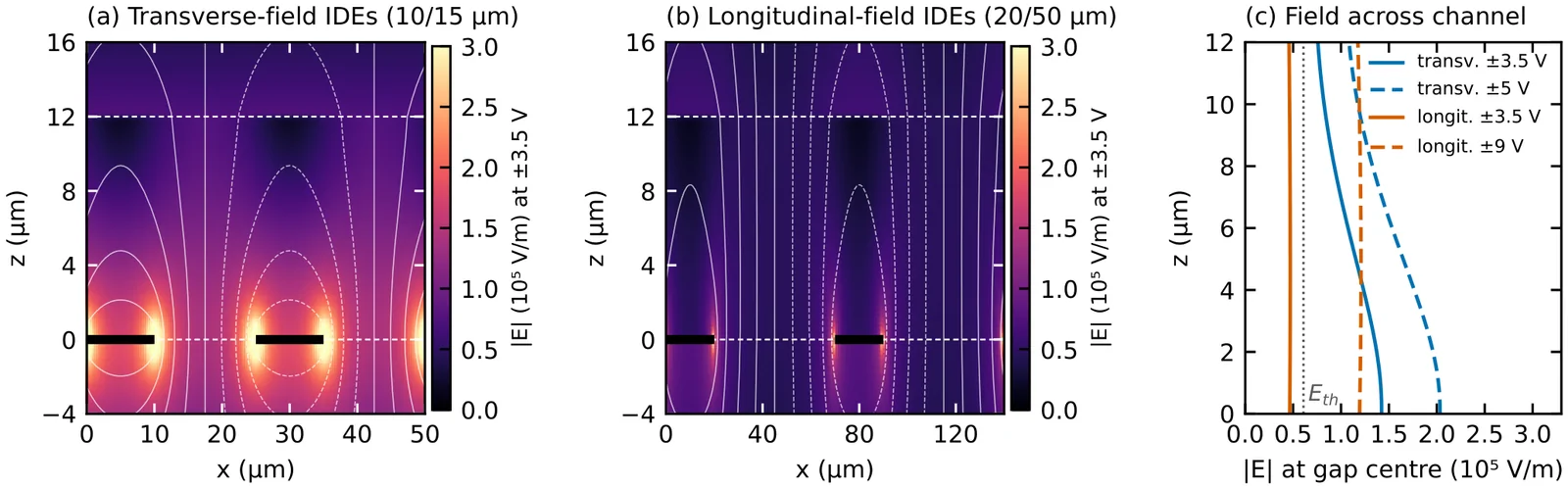
Unscreened electrostatic reference field of the IDE arrays, computed for an idealized periodic glass/ITO/5CB/PDMS stack. (**a**) Transverse-field geometry (electrode width 10 μm, gap 15 μm) and (**b**) longitudinal-field geometry (width 20 μm, gap 50 μm): Field magnitude |E| at $U_{0}=3.5$ V (color scale) with equipotential lines (white); the black bars mark the ITO fingers at z=0 and the dashed lines the boundaries of the 5CB layer. (**c**) Field magnitude at the gap center as a function of the height *z* in the channel for the experimental voltages; the dotted line marks the uniform-layer reorientation field scale $E_{\mathrm{th}}$ for a 12 μm homeotropic layer.

## Data Availability

The original contributions presented in this study are included in the article/Supplementary Material. Further inquiries can be directed to the corresponding author.

## Supplementary Materials

The following are available online at https://www.mdpi.com/article/10.3390/ma19204355/s1: Movie S1: Reshaping of a flowing dowser domain by an in-plane electric field applied perpendicular to the flow (illustrating the protocol in Figure 2a); the transient isotropic regions at about 3 and 9 s are produced by preparation laser pulses; Movie S2: Electric-field-induced nucleation of a single dowser domain under pulsating flow (related to Figure 3b); Movie S3: Manipulation of dowser domains by an in-plane electric field applied along the channel under alternating flow (related to Figure 4b); the dark circular feature visible at the beginning and at about 8 s is the transient isotropic region created by the IR laser pulse used to generate a fresh dowser domain upstream of the recorded area; Movie S4: Electric-field-induced nucleation of chiral $B^{★}$ domains in constant-pressure-driven flow (related to Figure 5b), with successive packets emitted approximately every 2 s.

## Appendix A. Electrostatic Field of the Interdigitated Electrodes

To estimate the geometry-dependent unscreened reference field, we solved the two-dimensional electrostatic problem ∇·(ε∇ϕ)=0 in the plane normal to the electrode fingers for one period $\Lambda =2(w_{e}+g)$ of each electrode array, using an idealized periodic section of the device stack with relative permittivities: glass substrate (ε=4.7) below the electrode plane, the 12 μm 5CB layer (treated in the isotropic approximation with the average permittivity $\bar{\varepsilon }\approx 11$), and PDMS (ε=2.7) above. The ITO fingers were modeled as infinitely thin electrodes in the plane z=0, held at $\pm U_{0}/2$, so the potential difference between neighboring electrodes is $U_{0}$; the thin DMOAP layer was omitted from this electrostatic approximation. The computational domain was periodic in *x* and closed by grounded boundaries 60 μm below and above the stack. The equation was discretized with a flux-conservative five-point finite-difference scheme on a uniform grid (Δx=0.5μm, Δz=0.2μm) and solved with a sparse direct solver (Python 3.11.15 with NumPy 2.4.4 and SciPy 1.17.1); since the problem is linear, it was solved for $U_{0}=1$ V and the fields were scaled to the experimental voltages. Halving the grid spacing or doubling the distance to the grounded boundaries changed the field in the nematic layer by less than 1.2%, and the computed field agrees within 0.6% with the exact Fourier-mode solution for the layered medium obtained from the computed electrode-plane potential. Figure A1a,b shows the resulting field magnitude and equipotential lines for the two geometries. The model neglects dielectric anisotropy, mobile ions, electrode charging, electrochemical reactions, finite-array end effects, and the feedback of director reorientation on the field; it therefore does not predict the time-dependent field during the 1 Hz drive or the field at the array ends where domains nucleate.

For a periodic array of alternating electrodes, the potential at the electrode plane has the spatial period $\Lambda =2(w_{e}+g)$, so that in a homogeneous medium, its fundamental Fourier component decays into the sample over the characteristic length Λ/2π, i.e., ≈8μm for the transverse-field IDEs and ≈22μm for the longitudinal-field IDEs. In this layered electrostatic model, the decay is substantially slower, because the high-permittivity nematic layer sandwiched between low-permittivity glass and PDMS channels the field lines: The computed gap-center field of the transverse-field IDEs decays with an effective length of ≈16μm and remains above the dielectric reorientation threshold $E_{\mathrm{th}}\approx 6\times 10^{4}\;Vm^{-1}$ over the entire channel height already at $U_{0}=3.5$ V, while the field of the longitudinal-field IDEs is nearly uniform across the channel height and crosses $E_{\mathrm{th}}$ between $U_{0}=3.5$ V (below threshold) and $U_{0}=9$ V (above threshold), as shown in Figure A1c. This comparison is qualitatively consistent with reshaping at ±3.5 V and nucleation at ±9 V in the longitudinal configuration. The uniform-layer threshold is a reference scale, not a local nucleation criterion for a spatially nonuniform field acting on a flowing dowser texture. The vertical field components are strongest above the electrode edges, where they are comparable to or exceed the in-plane components–the classical fringing-field situation whose role in defect generation has been analyzed in Ref. [52].
