# Supplementary material for: Shaping Nematic Liquid Crystal Flow States with In-Plane Electric Fields
Source: Materials (Basel). 2026 Oct 16;19(20):4355. doi: 10.3390/ma19204355 (PMC13707002; doi:10.3390/ma19204355)
Supplement: Supplementary file 1 [file materials-19-04355-s001.zip › materials-4576130-supplementary.pdf]

Supplementary Materials for
Shaping Nematic Liquid Crystal Flow States with In-Plane Electric Fields

Tadej Emeršič[1] and Uroš Tkalec[2,3,4*]

[1] Department of Physics and Materials Science, Faculty of Science, Technology and Medicine, University of Luxembourg, 162a avenue de la Faiencerie, L-1511 Luxembourg, Luxembourg

[2] Institute of Biophysics, Faculty of Medicine, University of Ljubljana, Vrazov trg 2, 1000 Ljubljana, Slovenia

[3] Department of Condensed Matter Physics, Jožef Stefan Institute, Jamova 39, 1000 Ljubljana, Slovenia

[4] Faculty of Natural Sciences and Mathematics, University of Maribor, Koroška 160, 2000 Maribor, Slovenia

[*] Corresponding author: uros.tkalec@mf.uni-lj.si

Description of Movies

Movie S1

Reshaping of a flowing dowser domain by an in-plane electric field applied perpendicular to the flow (illustrating the protocol in Figure 2a); the transient isotropic regions at about 3 and 9 s are produced by preparation laser pulses.

Additional details. A square-wave AC signal of ±3.5 V at 1 Hz changes the shape and birefringent colors of the domain as it passes through the electrode region. After leaving the region, the domain recovers its unperturbed shape and continues to grow. The before/after velocity comparison uses the intervals 4.0–5.0 and 8.0–8.8 s and excludes the later laser pulse.

Movie S2

Electric-field-induced nucleation of a single dowser domain under pulsating flow (related to Figure 3b).

Additional details. A square-wave AC signal of ±5.0 V at 1 Hz is applied perpendicular to the flow, while the flow velocity is sinusoidally modulated between 0 and 80 $\mu\mathrm{m\,s^{-1}}$ with a period of 5 s. Under these conditions, the electric field nucleates a single elongated dowser domain, which subsequently evolves with the pulsating flow.

Movie S3

Manipulation of dowser domains by an in-plane electric field applied along the channel under alternating flow (related to Figure 4b); the dark circular feature visible at the beginning and at about 8 s is the transient isotropic region created by the IR laser pulse used to generate a fresh dowser domain upstream of the recorded area.

Additional details. The flow velocity is varied between -60 and $+60$ $\mu\mathrm{m\,s^{-1}}$ with a period of 4 s, causing the reorientation of the entrapped director field each time the flow direction is reversed. Flow reversal is accompanied by the formation of surface point defects and realignment fronts, while the applied in-plane electric field further perturbs and reshapes the director configuration within the dowser domains.

Movie S4

Electric-field-induced nucleation of chiral $B^\star$ domains in constant-pressure-driven flow (related to Figure 5b), with successive packets emitted approximately every 2 s.

Additional details. At a flow velocity of 80 $\mu\mathrm{m\,s^{-1}}$, an in-plane electric field applied along the microfluidic channel (±9.0 V, 1 Hz) induces the formation of a steady sequence of chiral $B^\star$ domains, with approximately one domain emitted every 2 s. Seven successive packets give six intervals of 1.96–2.04 s (mean 2.00 s).

The movies play at 25 frames per second in real time.
